# Editorial: Community series in emerging insights in controlling autoimmunity, volume II

**DOI:** 10.3389/fimmu.2025.1613858

**Published:** 2025-06-02

**Authors:** Lorenza Bruno, Dimitrios Bogdanos, Maria Maslinska, Carlo Perricone

**Affiliations:** ^1^ Section of Rheumatology, Department of Medicine and Surgery, University of Perugia, Perugia, Italy; ^2^ Department of Rheumatology and Clinical Immunology, Faculty of Medicine, University of Thessaly, Larissa, Greece; ^3^ Early Arthritis Clinic of the National Institute of Geriatrics, Rheumatology and Rehabilitation, Warsaw, Poland

**Keywords:** autoimmunity, IL-17, Th17, breg, lymphocytes

Autoimmunity and autoimmune diseases evolve when mechanisms of self-tolerance fail, allowing the expansion of auto-reactive B and T lymphocytes and the continuous secretion of pro-inflammatory cytokines. For many years, our main attention was directed towards a better understanding of the complexity of both humoral and cellular responses in autoimmunity, specifically, the roles of autoantibodies and Th1/Th17 or Th2-related pro-inflammatory cytokines in different autoimmune diseases (1). This was subsequently followed by the development of anti-inflammatory therapies, largely aimed at blocking these cytokines with relevant monoclonal antibodies. Without neglecting the role of pro-inflammatory responses in the pathogenesis of autoimmune diseases, there has recently been an increased focus on assessing the importance of the regulatory system, preventing the development of autoimmune diseases. Initially, T regulatory cells (Tregs, comprising cells approximately 10% of all CD4+ T cells) were considered to be the only regulatory cells, whose role is to prevent the expansion of auto-reactive T and B lymphocytes, natural killer cells, and dendritic cells (2). This is achieved by the ability of these cells, upon specific activation, to secrete inhibitory cytokines, such as interleukin-10 (IL-10), transforming growth factor β (TGF-β) and IL-35. The expression of the inhibitory molecule by down-regulating co-stimulatory molecules on effector T lymphocytes and dendritic cells. Cytotoxic T cell antigen 4 (CTLA-4) on Tregs enables a cell-to-cell form of regulation by down-regulating co-stimulatory molecules on effector T lymphocytes and dendritic cells. Cytotoxic T cell antigen 4 on Tregs allows for a cell-to-cell regulation, as it down-regulates co-stimulatory molecules on effector T lymphocytes and dendritic cells. As well as CTLA-4 on Tregs enables this cell-to-cell regulation by downregulating co-stimulatory molecules on effector T lymphocytes and dendritic cells. More recently, B regulatory cells (Bregs) have also been indicated as an essential feature of regulatory mechanisms. B regulatory cells have no specific transcription factor such as FoxP3 (forkhead box P3) or specific membrane molecules identified. However, they do secrete inhibitory cytokines, such as IL-10 and IL-35, in a specific antigen-dependent manner, and they also secrete blocking IgG4, which plays a role in preventing allergic diseases (3).

Understanding the role of regulatory cells in different autoimmune diseases may lead to a response towards tailored treatment of these diseases, and molecules in different autoimmune diseases may lead to a response towards tailored treatment of these diseases, which are burdened by high morbidity and mortality. In this Research Topic of Frontiers in Immunology, a special focus was given on the role of Th17 cells. Indeed, it is well established that inflammatory cytokines and T cell subpopulations play a crucial role in inflammation and disease progression; however, findings regarding their specific roles remain largely inconsistent. The involvement of the Th17/IL-17 axis in the pathogenesis of chronic autoimmune diseases is well established. In psoriasis, circulating T follicular helper cells (cTfh) and circulating T helper cells (cTph) closely interact with the interleukin 17 (IL-17) axis. Murine models have shown that elevated expression of molecules within the IL-23/IL-17 axis enhances Tfh-mediated functions. Considering the frequent use of anti-IL-17 neutralizing agents in the treatment of psoriasis, Tsiogkas et al. investigated the impact of this biologic treatment on human T cells, demonstrating a reduction in the subpopulations of cTfh and cTph cells. Interleukin 17-producing cells have also been detected in the kidneys of lupus-prone mice and patients with lupus nephritis (LN), suggesting that IL-17 is implicated in the pathogenesis of organ damage. However, several studies from the literature have provided conflicting data regarding the imbalance between Th17 and Treg cells. In this context, the work by Huang et al. provides a robust meta-analytic framework that underscores the imbalance between Th17 cells and Tregs in systemic lupus erythematosus (SLE). Their results highlight elevated levels of Th17 cells and pro-inflammatory cytokines, such as IL-17, IL-21, and IL-6, alongside a reduction in the anti-inflammatory cytokine TGF-β, in SLE patients compared to healthy controls. This provides an explanation for the elevated Th17/Treg ratio observed in SLE. Interestingly, this Th17/Treg imbalance appears to correlate with disease activity and organ-specific manifestations such as lupus nephritis (LN). Elevated Th17 responses, particularly in patients with renal involvement, point to the potential utility of Th17-associated cytokines as biomarkers for disease severity. Moreover, this immune dysregulation varies with age, sex, and glucocorticoid use, adding layers of complexity to both diagnosis and treatment strategies. These findings reinforce the notion that SLE pathogenesis is not merely a consequence of immune activation but rather an intricate failure of regulatory mechanisms that normally suppress autoimmunity.

Complementing these immunological insights, Horisberger et al. (4) utilized transcriptomic data from the AMP (Accelerating Medicines Partnership) SLE consortium to redefine patient stratification using B cell gene signatures. By identifying three distinct B cell-related molecular subtypes across whole blood, peripheral blood mononuclear cells (PBMCs), and kidney tissue, they illustrate the potential of transcriptional profiling in advancing personalized medicine. These subtypes—characterized by signatures of naïve B cells, antibody-producing cells, and a blend of both—offer a window into the cellular contributors of disease phenotype and therapeutic response. Notably, antibody-producing cell signatures were most enriched in kidney tissue, reinforcing the importance of B cell-driven immunity in LN pathogenesis.

Although IL-17A is not considered a canonical cytokine in SLE, its role in the pathogenesis and activity of the disease is well established. Recently, Eiza et al. demonstrated a significant increase in the expression of pro-inflammatory cytokines, including IL-17A and IFN-gamma, mediated by soluble CD72 (sCD72). Elevated sCD72 levels were found in the serum of patients with autoimmune conditions such as SLE and primary Sjögren’s syndrome. Furthermore, interaction among T cells may represent a signaling pathway involved in the development of immune-mediated diseases and may represent a potential therapeutic target. In addition to IL-17, other inflammatory mediators play a role in these conditions. For example, in chronic spontaneous urticaria (CSU), T cell/mast cell proximity in skin lesions is associated with increased IL-17 expression. Since the mechanisms driving T cell/mast cell co-localization remain unclear, Mubariki et al. investigated whether chemokines expressed in lesional CSU skin contribute to T cell/mast cell proximity. Their findings suggest that the close association of T cells and mast cells in the skin of patients with severe CSU may be partly driven by elevated expression of CCR5 and CCL3. Therefore, the interaction between CCL3 and CCR5 could represent an additional target for future therapeutic strategies in CSU. Finally, to better understand the pathogenesis of these conditions and develop more targeted and effective treatments, artificial intelligence and advanced genomics techniques are playing an increasingly important role. For instance, Mou et al. used machine learning (ML) algorithms to analyze renal biopsy data, identifying key genes as diagnostic markers with high predictive accuracy. They applied ML to predict LN with high accuracy. By evaluating twelve ML algorithms in tandem with Non-negative Matrix Factorization (NMF) for feature extraction, the authors develop a five-gene panel capable of predicting LN with an AUC of 0.958. This model also exhibits a significant correlation with histological classes of nephritis and immune infiltration scores. Importantly, the genes identified—such as TYROBP and FCER1G—are enriched in monocyte/macrophage pathways, further confirming the contribution of innate immunity in LN.

Together, these three studies demonstrate a powerful convergence of data-driven and hypothesis-driven research in autoimmune disease. They collectively emphasize the need for system-level analyses that incorporate cytokine profiling, immune cell subset quantification, and transcriptomic modeling to refine our understanding of SLE. Such integrative approaches are essential not only for identifying reliable biomarkers but also for tailoring immunomodulatory treatments to patient-specific disease drivers.

Moving forward, bridging cellular immunology with computational biology will be key to addressing the unmet clinical needs in SLE. The translation of these insights into clinical practice - whether through predictive ML tools or molecular subtyping - offers a promising avenue toward precision medicine in rheumatology. These biomarkers offer a promising opportunity to thereby enhancing early diagnosis and treatment and reducing the need for invasive interventions.

These recent studies have provided crucial insights into the underlying immunological pathogenesis of autoimmune and inflammatory diseases. The central role of the Th17/IL-17 axis, the involvement of specific cytokines and T cell subpopulations, and the involvement of specific cytokines and T cell subpopulations, is a common thread across these diseases. This highlights the delicate balance of immune responses that can lead to the onset and progression of disease. Further elucidating the interactions between cytokines, receptors, and immune cells, such as IL-17, CCR5, and sCD72, and their signaling pathways not only will enhance our understanding of the mechanisms underlying these diseases but also may allow to identify novel therapeutic opportunities. For example, the discovery of the sCD72-CD6 axis represents a promising new target for therapeutic intervention in autoimmune disorders. Furthermore, advanced technologies to identify novel biomarkers and predict disease progression could revolutionize early diagnosis and personalized patient management, ultimately transforming the clinical approach to these complex diseases.
